# Supplementary material for: Promoting abnormal grain growth in Fe-based shape memory alloys through compositional adjustments
Source: Nat Commun. 2019 May 28;10:2337. doi: 10.1038/s41467-019-10308-8 (PMC6538750; doi:10.1038/s41467-019-10308-8)
Supplement: Supplementary file 1 — Supplementary Information [file 41467_2019_10308_MOESM1_ESM.pdf]

Supplementary Information

Promoting abnormal grain growth in Fe-based shape memory alloys through compositional adjustments

Vollmer et al.

Supplementary Discussion

Grain size distributions after solution annealing and after a single cyclic heat treatment (CHT)

In order to investigate the influence of abnormal grain growth (AGG) on the grain size distribution of samples with different chemical compositions in detail, one sample of each composition and heat treatment condition, i.e. after a solution annealing at 1225 °C for 1h and after one CHT cycle (cf. Supplementary Figure 1a), was evaluated by means of grain area analyses. Supplementary Figure 2 shows the frequency and the total area fraction of different grain size classes for (a) Fe–Mn–Al–Ni, (b) Fe–Mn–Al–Ni–Ti and (c) Fe–Mn–Al–Ni–Cr. Comparing the solution treated condition of the different chemical compositions, it is obvious that most of the total area fraction of all samples is covered by grains with an area between 0.1 – 1 mm^2. In contrast, after a single CHT AGG occurred in Fe–Mn–Al–Ni and Fe–Mn–Al–Ni–Ti as it is obvious from the shift of the grain size distribution to higher values, whereas the grain size distribution of Fe–Mn–Al–Ni–Cr was found to remain almost the same, due to the absence of AGG. Assuming that grains with an area larger than 1 mm^2 are abnormally grown grains, it can be seen that in Fe–Mn–Al–Ni about 77% of the total area is covered by such grains, whereas in Fe–Mn–Al–Ni–Ti more than 98% of the total area is covered by abnormally grown grains. Moreover, no grains with an area larger than 10 mm^2 were obtained in Fe–Mn–Al–Ni, whereas grains with an area of more than 10 mm^2 provide for the highest frequency in Fe–Mn–Al–Ni–Ti.

On the influence of subgrain structures on the pseudoelastic behavior

The microstructural appearance of single crystals obtained by AGG through a CHT can be divided into two groups: One without subgrain structures where grain growth occurred during the final solution annealing step and one with subgrain structures where the grain was already present before the final solution annealing. In order to investigate the influence of subgrain structures on the martensitic transformation, a compression sample was wire-cut by EDM from the same single crystalline Fe–Mn–Al–Ni–Ti bar, from which the sample shown in Figure 8c was extracted from. Care was taken that the sample was wire-cut from a region with a high density of subgrain structures. Before testing the sample

was solution annealed at 1225 °C for 30 min and subsequently quenched in 80 °C warm water to avoid influences imposed by the EDM process. This particular condition was chosen due to the expected high average misorientation of the subgrains (about 3°). As it was shown by Kusama et al. [1] for Cu–Al–Mn, low temperature cycles before the last solution annealing lead to increased subgrain misorientation. It is obvious from the stress-strain curve shown in Supplementary Figure 3a that the Fe–Mn–Al–Ni–Ti sample with a high density of subgrain structures shows a higher critical stress for martensitic transformation, a larger stress hysteresis and a lower recoverability in comparison with the sample shown in Figure 8c at 4% applied strain. The optical micrograph of the surface of the sample in the loaded condition shown in Supplementary Figure 3b reveals pronounced topography within the martensitic plates and in direct vicinity of the martensitically transformed area. Moreover, subgrain structures are clearly visible within the martensite plates and in some subgrains additional martensite variants can be seen. After unloading, pronounced topography remains in areas where the martensitic transformation occurred in the previous loading step as it is shown in Supplementary Figure 3c. These topography changes can be clearly linked to the residual strain seen in the stress-strain curve.

On the functional fatigue behavior of Fe–Mn–Al–Ni–Ti single crystals grown by AGG

Two compression samples were wire-cut by EDM from the single crystalline Fe–Mn–Al–Ni–Ti bar mentioned before (Figure 8c and Supplementary Figure 3). One of the samples was extracted from an area free of subgrain structures and one from an area with subgrain structures. Afterwards samples were solution annealed at 1225 °C for 30 min and subsequently quenched in 80 °C warm water. Results from constant amplitude functional fatigue tests up to 4% applied strain are shown in Supplementary Figure 4. The samples were cyclically loaded for 20 and 10 times, respectively. The sample without subgrain structures reveals excellent pseudoelasticity in the first cycle, i.e. minor hysteresis width and full strain recovery. In further cycles, functional fatigue sets in and the degree of recoverability is decreased. The stress level at the end of the loading plateau increases during cycling. It is very likely that the increased stress is related to the transformation of areas which were previously not transformed, as it was already reported in Fe–Mn–Al–Ni [2,3]. However, an in-depth analysis of the degradation mechanisms of Fe–Mn–

Al–Ni–Ti in comparison with the degradation mechanisms found in Fe–Mn–Al–Ni [2,3] is clearly out of scope of the current study and, thus, will be subject of future work. The sample with a high fraction of subgrain structures again clearly reveals higher stresses for martensitic transformation, larger widths of the stress hysteresis and a lower recoverability as compared to the sample without subgrain structures. It is expected that the faster functional degradation is related to the interaction between the martensite and the subgrain structures as shown in Supplementary Figure 3. Finally, it is obvious that the functional properties are completely lost after 10 cycles in the sample with subgrain structures, whereas irreversible strain of the sample without subgrain structures is only 0.65% after 10 cycles.

On the abnormal grain growth behavior in a 300 mm Fe–Mn–Al–Ni–Ti bar

One Fe–Mn–Al–Ni–Ti bar with a length of 300 mm and a diameter of 6.3 mm was subjected to the CHT process shown in Supplementary Figure 1d in order to reveal that the growth of single crystals can be extended to samples with dimensions exceeding 100 mm, while the 100 mm bars served as the basis for the experiments in the present work. As it is shown in the Supplementary Figure 7, two grains with a size of 220 mm and 80 mm, respectively, were revealed by optical microscopy and X-ray diffraction measurements. From the shape of the grain boundary highlighted in (b) it is deduced that the grain of 220 mm grew consuming the (remaining) grain of 80 mm before quenching. Probably it is possible to further increase the maximum size of the single crystals by changing the heat treatment procedure, e.g. by integrating larger dwell times in the single phase region before quenching, applying lower temperatures in the two phase region, a higher number of heat treatment cycles, different temperature ramps, or by using thermo-mechanical treatments for texturing before AGG. Further analysis of this matter will be subject of future studies.

Supplementary Figures

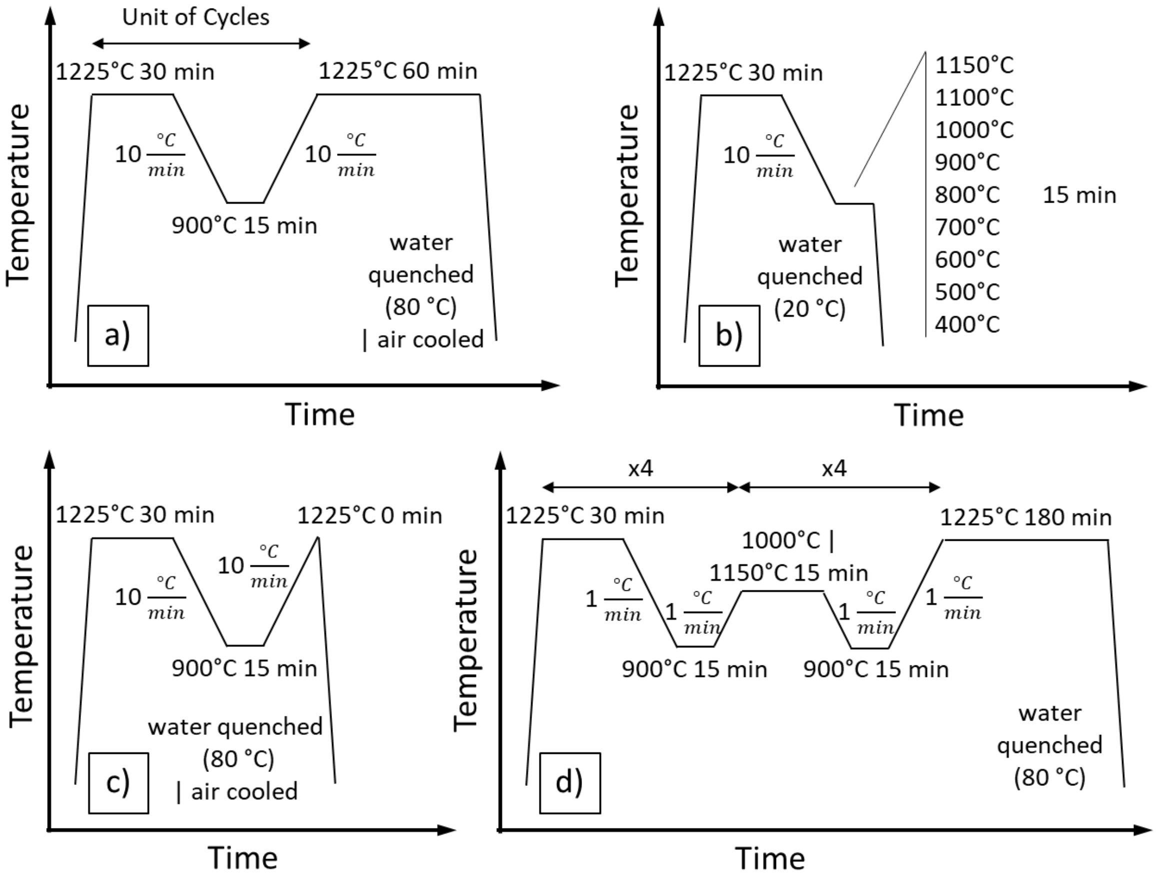

Supplementary Figure 1: Heat treatment procedures used in the current study. Dog-bone shape tension samples of Fe–Mn–Al–Ni, Fe–Mn–Al–Ni–Ti and Fe–Mn–Al–Ni–Cr were used for procedures (a), (b) and (c), respectively. Fe–Mn–Al–Ni and Fe–Mn–Al–Ni–Ti bars were used in (d). Results obtained upon CHT shown in (a) were used for Figures 1 and 2, CHT shown in (b) for Figures 3 and 4, CHT shown in (c) for Figures 5, 6 and 7 and CHT shown in (d) for Figure 8, respectively.

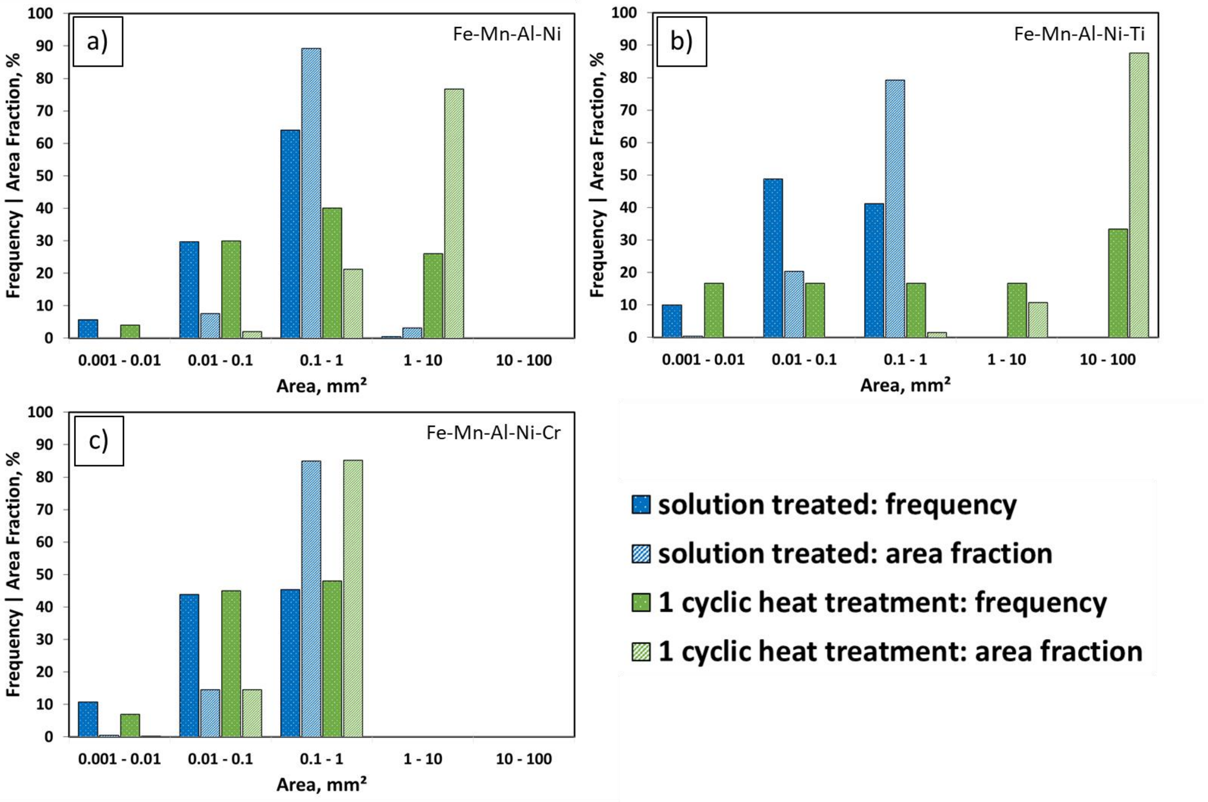

Supplementary Figure 2: Grain size distributions of representative samples of Fe–Mn–Al–Ni, Fe–Mn–Al–Ni–Ti and Fe–Mn–Al–Ni–Cr after solution annealing and after a single cyclic heat treatment. Frequencies and area fractions of the different area classes of grains are shown for (a) Fe–Mn–Al–Ni, (b) Fe–Mn–Al–Ni–Ti and (c) Fe–Mn–Al–Ni–Cr. Results for the solution treated condition (1225°C 1h) are colored blue, whereas results after a single cyclic heat treatment (cf. Supplementary Figure 1a) are colored green.

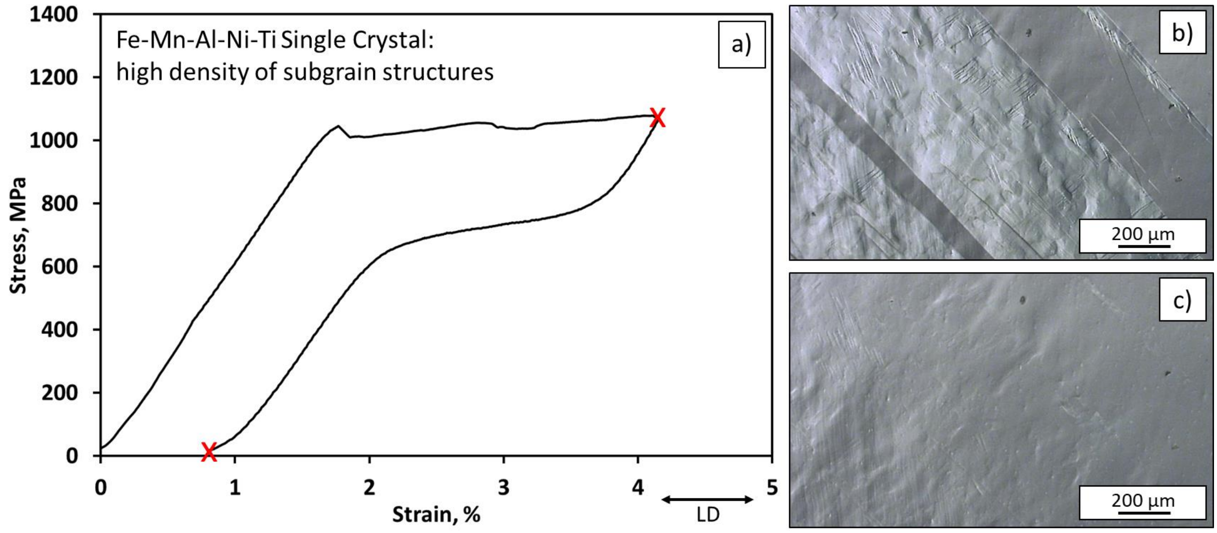

Supplementary Figure 3: In situ investigations on the influence of subgrain structures on the pseudoelastic behavior of Fe–Mn–Al–Ni–Ti single crystals obtained by abnormal grain growth. (a) Stress – strain curve up to 4% applied strain of a single crystalline Fe–Mn–Al–Ni–Ti compression sample with a high density of subgrain structures. Optical micrographs show the topography evolution on the surface upon loading (b) and unloading (c) of the sample (highlighted by red crosses in the pseudoelastic curve). The loading direction (LD) is related to the optical micrographs. The sample has the same orientation as the sample shown in Figure 8c.

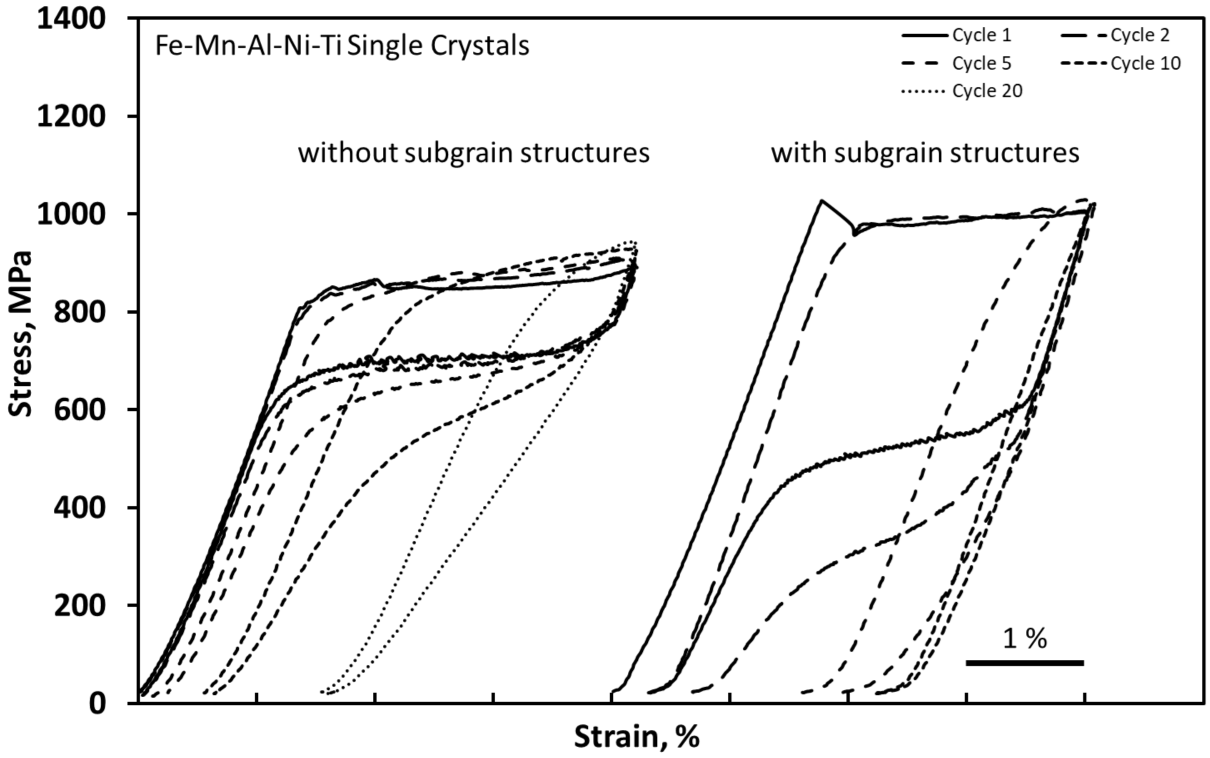

Supplementary Figure 4: Functional fatigue tests of single crystalline Fe–Mn–Al–Ni–Ti compression samples with different fractions of subgrain structures. Stress – strain curves up to 4% applied strain from a sample without (left) and with (right) subgrain structures. Both samples were wire cut by EDM from the same single crystalline Fe–Mn–Al–Ni–Ti bar obtained by abnormal grain growth. The sample orientation with respect to the loading direction is the same as shown in the inset of Figure 8c.

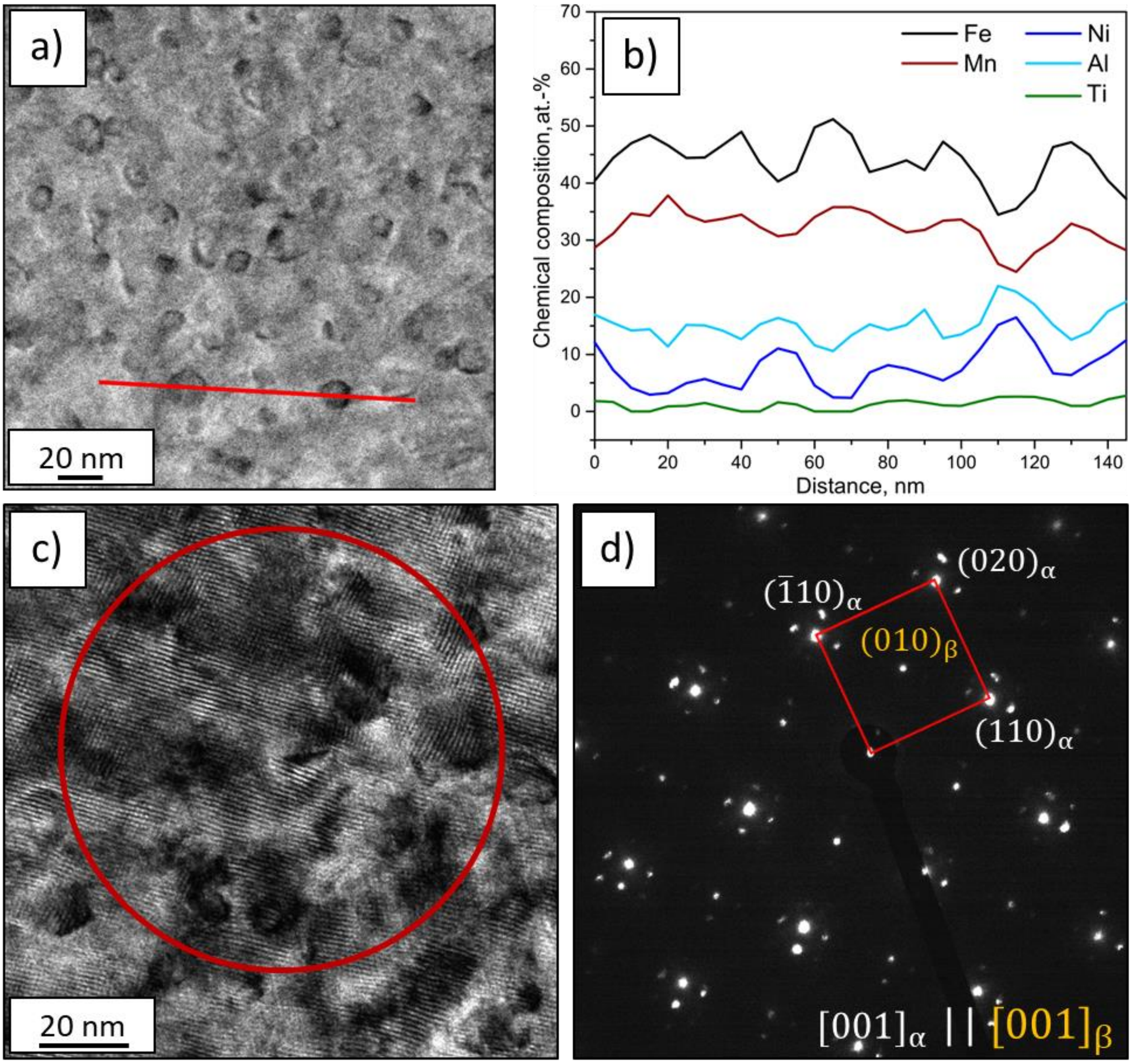

Supplementary Figure 5: Investigation of orientation relationship and chemical composition of β precipitates. (a) Scanning transmission electron microscopy image of the Fe–Mn–Al–Ni–Ti compression sample (FIB sample extracted from sample characterized in Figure 8c). The red line indicates the lateral position of the energy dispersive X-ray spectroscopy line-scan shown in (b). Transmission electron microscopy image (c) with corresponding selected area diffraction pattern (d) revealing the orientation relationship $[001]_\alpha \parallel [001]_\beta$. Satellite reflections in (d) are probably caused by Moiré contrasts. The red circle on the left indicates the region from which the SAED pattern was recorded and on the right, the red square marks the reciprocal bcc cell.

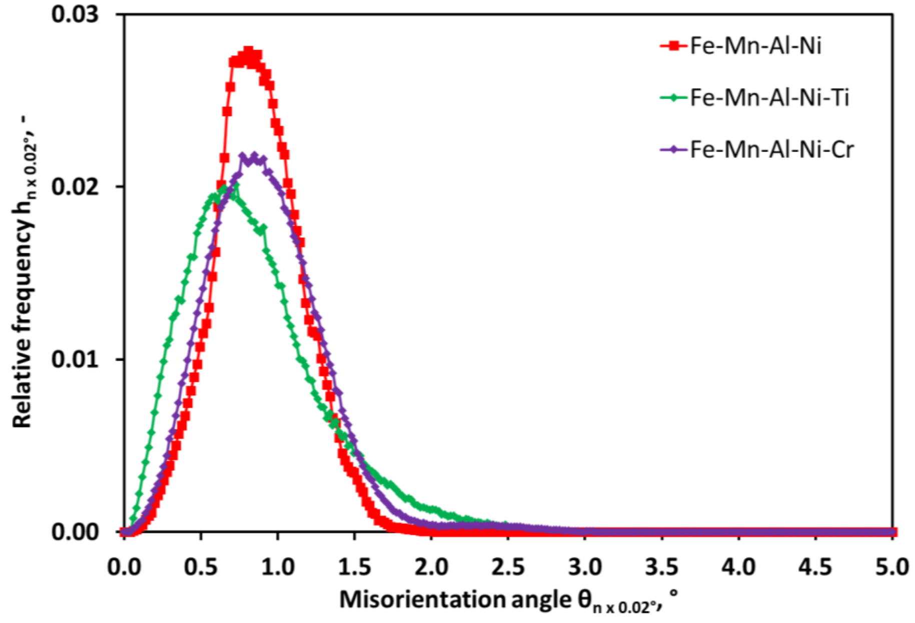

Supplementary Figure 6: Evaluation of the mean average misorientation of the subgrains. Relative frequency of the misorientations ($h_{n\,x\,0.02°}$) of subgrains as a function of the misorientation angle ($\theta_{n\,x\,0.02°}$) for the three different chemical compositions considered.

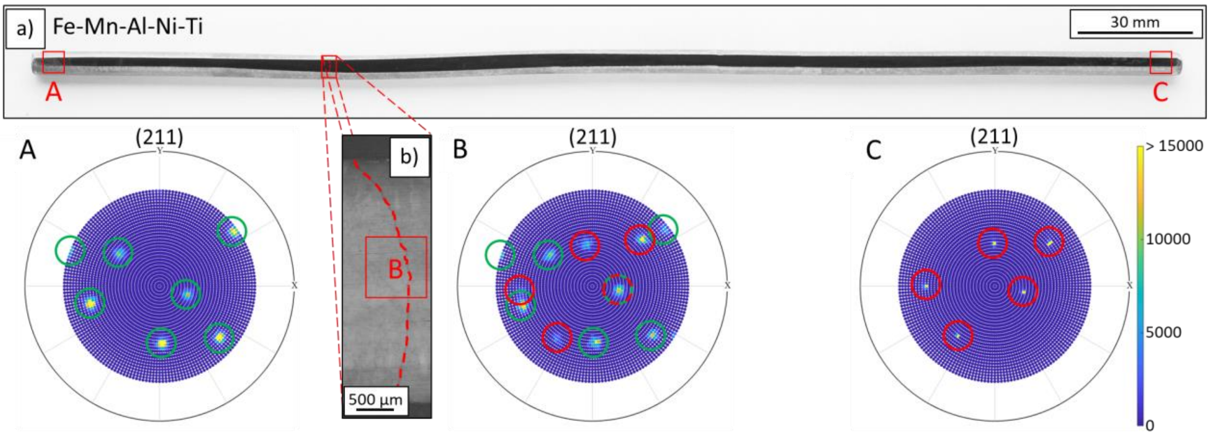

Supplementary Figure 7: Abnormal grain growth in a 300 mm Fe–Mn–Al–Ni–Ti bar. (a) Overview image of the Fe–Mn–Al–Ni–Ti bar with a length of 300 mm and a diameter of 6.3 mm upon the CHT procedure shown in Supplementary Figure 1d. (b) OM micrograph highlighting one single grain boundary in the sample. The grain boundary shown in (a) and (b) is highlighted by red dashed lines. (211) pole figures were taken from the areas highlighted by the red squares in (a) and (b), respectively. Poles of the left grain are marked by green circles and poles of the right grain are marked by red circles.

Supplementary Tables

Material	Heat Treatment Procedure	AGG migration rate (exp.) (m s^{-1})	AGG migration rate (calc.) (m s^{-1})	ΔG_{total} (J mol^{-1})	ΔG_s (J mol^{-1})	ΔG_h (J mol^{-1})	C_s	C_n	C_a	γ_s (J m^{-2})	γ_h (J m^{-2})	θ (°)	θ_h (°)	V (m^3 mol^{-1})	r_s (m)	r_n (m)	r_a (m)	D^{gb} (m^2 s^{-1})	δ (m)	R (J mol^{-1} K^{-1})	T (K)
Cu-Al-Mn*	500 °C – 800 °C | $r_a = r_n$	1.60 x 10^{-5}	3.73 x 10^{-6}	0.031	0.031	0	1.5	1	1	0.082	0.595	0.46	15	7.6 x 10^{-6}	30 x 10^{-6}	400 x 10^{-6}	400 x 10^{-6}	5.36 x 10^{-10}	5 x 10^{-10}	8.314	1073.15
	500 °C – 800 °C | $r_a \gg r_n$		5.77 x 10^{-6}	0.048	0.031	0.017	1.5	1.5	1	0.082	0.595	0.46	15	7.6 x 10^{-6}	30 x 10^{-6}	400 x 10^{-6}	∞	5.36 x 10^{-10}	5 x 10^{-10}	8.314	1073.15
Fe-Mn-Al-Ni**	900 °C – 1200 °C | $r_a = r_n$	2.50 x 10^{-6}	1.70 x 10^{-6}	0.029	0.029	0	1.5	1	1	0.153	0.617***	1	15	7.366x 10^{-6}****	57.5 x 10^{-6}	254 x 10^{-6}	254 x 10^{-6}	5.33 x 10^{-10}	7.5 x 10^{-10}	8.314	1473.15
	900 °C – 1200 °C | $r_a \gg r_n$		3.26 x 10^{-6}	0.056	0.029	0.027	1.5	1.5	1	0.153	0.617***	1	15	7.366x 10^{-6}****	57.5 x 10^{-6}	254 x 10^{-6}	∞	5.33 x 10^{-10}	7.5 x 10^{-10}	8.314	1473.15
Fe-Mn-Al-Ni (this study)	900 °C – 1225 °C | $r_a = r_n$		1.60 x 10^{-6}	0.028	0.028	0	1.5	1	1	0.139	0.617***	0.88	15**	7.366x 10^{-6}****	54.6 x 10^{-6}	327 x 10^{-6}	327 x 10^{-6}	5.33 x 10^{-10}**	7.5 x 10^{-10}**	8.314	1498.15
	900 °C – 1225 °C | $r_a \gg r_n$		2.80 x 10^{-6}	0.049	0.028	0.021	1.5	1.5	1	0.139	0.617***	0.88	15**	7.366x 10^{-6}****	54.6 x 10^{-6}	327 x 10^{-6}	∞	5.33 x 10^{-10}**	7.5 x 10^{-10}**	8.314	1498.15
Fe-Mn-Al-Ni-Ti (this study)	900 °C – 1225 °C | $r_a = r_n$	1.84 x 10^{-5}	7.42 x 10^{-6}	0.130	0.130	0	1.5	1	1	0.137	0.617***	0.86	15**	7.366x 10^{-6}****	11.6 x 10^{-6}	241 x 10^{-6}	241 x 10^{-6}	5.33 x 10^{-10}**	7.5 x 10^{-10}**	8.314	1498.15
	900 °C – 1225 °C | $r_a \gg r_n$		9.04 x 10^{-6}	0.158	0.130	0.028	1.5	1.5	1	0.137	0.617***	0.86	15**	7.366x 10^{-6}****	11.6 x 10^{-6}	241 x 10^{-6}	∞	5.33 x 10^{-10}**	7.5 x 10^{-10}**	8.314	1498.15
Fe-Mn-Al-Ni-Cr (this study)	900 °C – 1225 °C | $r_a = r_n$		2.56 x 10^{-6}	0.045	0.045	0	1.5	1	1	0.143	0.617***	0.92	15**	7.366x 10^{-6}****	35.3 x 10^{-6}	332 x 10^{-6}	332 x 10^{-6}	5.33 x 10^{-10}**	7.5 x 10^{-10}**	8.314	1498.15
	900 °C – 1225 °C | $r_a \gg r_n$		3.74 x 10^{-6}	0.065	0.045	0.021	1.5	1.5	1	0.143	0.617***	0.92	15**	7.366x 10^{-6}****	35.3 x 10^{-6}	332 x 10^{-6}	∞	5.33 x 10^{-10}**	7.5 x 10^{-10}**	8.314	1498.15

* [1], ** [4], *** [5], **** [6]

Supplementary Table 1: AGG velocity and corresponding parameters used for calculation. Data for the first two materials were extracted from Kusama et al.[1] and Omori et al.[4]. Data for the other materials were evaluated in this study unless otherwise stated.

Supplementary References

1. Kusama, T. *et al.* Ultra-large single crystals by abnormal grain growth. *Nature communications* **8,** 354 (2017).
2. Vollmer, M. *et al.* Cyclic degradation in bamboo-like Fe–Mn–Al–Ni shape memory alloys — The role of grain orientation. *Scripta Materialia* **114,** 156–160 (2016).
3. Vollmer, M. *et al.* Cyclic Degradation Behavior of ⟨001⟩ -Oriented Fe–Mn–Al–Ni Single Crystals in Tension. *Shap. Mem. Superelasticity* **3,** 335–346 (2017).
4. Omori, T., Iwaizako, H. & Kainuma, R. Abnormal grain growth induced by cyclic heat treatment in Fe-Mn-Al-Ni superelastic alloy. *Materials & Design* **101,** 263–269 (2016).
5. Humphreys, F. J. & Hatherly, M. *Recrystallization and related annealing phenomena.* 2nd ed. (Elsevier, Amsterdam, Boston, 2004).
6. Omori, T. *et al.* Superelastic effect in polycrystalline ferrous alloys. *Science (New York, N.Y.)* **333,** 68–71 (2011).
