# Supplementary material for: Promoting abnormal grain growth in Fe-based shape memory alloys through compositional adjustments
Source: Nat Commun. 2019 May 28;10:2337. doi: 10.1038/s41467-019-10308-8 (PMC6538750; doi:10.1038/s41467-019-10308-8)
Supplement: Supplementary file 3 — Description of Additional Supplementary Files [file 41467_2019_10308_MOESM3_ESM.docx]

**Description of Additional Supplementary Files**

**Supplementary Movie 1**: Pseudoelastic bending test of a single crystalline Fe–Mn–Al–Ni–Ti bar obtained by abnormal grain growth. The heat treatment procedure shown in Supplementary figure 1d was used.
